# Actin Cytoskeleton Regulation by the Yeast NADPH Oxidase Yno1p Impacts Processes Controlled by MAPK Pathways

**DOI:** 10.3390/antiox10020322

**Published:** 2021-02-22

**Authors:** Manuela Weber, Sukanya Basu, Beatriz González, Gregor P. Greslehner, Stefanie Singer, Danusa Haskova, Jiri Hasek, Michael Breitenbach, Campbell W.Gourlay, Paul J. Cullen, Mark Rinnerthaler

**Affiliations:** 1 Department of Biosciences, University of Salzburg, 5020 Salzburg, Austria; manuela.weber@stud.sbg.ac.at (M.W.); gregor.greslehner@univie.ac.at (G.P.G.); Steffi_singer_14@gmx.de (S.S.); michael.breitenbach@sbg.ac.at (M.B.); 2 Department of Biological Sciences, State University of New York at Buffalo, Buffalo, NY 14260-1300, USA; basus@buffalo.edu (S.B.); beatrizg@buffalo.edu (B.G.); 3 Laboratory of Cell Reproduction, Institute of Microbiology of the Czech Academy of Sciences, Videnska 1083, 142 20 Prague 4, Czech Republic; danusa.haskova@biomed.cas.cz (D.H.); hasek@biomed.cas.cz (J.H.); 4 Kent Fungal Group, School of Biosciences, University of Kent, Kent CT2 9HY, UK; C.W.Gourlay@kent.ac.uk

**Keywords:** ROS, MAP kinase pathway, actin, osmotic stress, filamentous growth, invasive growth, pseudohyphal growth, pheromone response, apoptosis

## Abstract

Reactive oxygen species (ROS) that exceed the antioxidative capacity of the cell can be harmful and are termed oxidative stress. Increasing evidence suggests that ROS are not exclusively detrimental, but can fulfill important signaling functions. Recently, we have been able to demonstrate that a NADPH oxidase-like enzyme (termed Yno1p) exists in the single-celled organism *Saccharomyces cerevisiae*. This enzyme resides in the peripheral and perinuclear endoplasmic reticulum and functions in close proximity to the plasma membrane. Its product, hydrogen peroxide, which is also produced by the action of the superoxide dismutase, Sod1p, influences signaling of key regulatory proteins Ras2p and Yck1p/2p. In the present work, we demonstrate that Yno1p-derived H_2_O_2_ regulates outputs controlled by three MAP kinase pathways that can share components: the filamentous growth (filamentous growth MAPK (fMAPK)), pheromone response, and osmotic stress response (hyperosmolarity glycerol response, HOG) pathways. A key structural component and regulator in this process is the actin cytoskeleton. The nucleation and stabilization of actin are regulated by Yno1p. Cells lacking *YNO1* showed reduced invasive growth, which could be reversed by stimulation of actin nucleation. Additionally, under osmotic stress, the vacuoles of a ∆*yno1* strain show an enhanced fragmentation. During pheromone response induced by the addition of alpha-factor, Yno1p is responsible for a burst of ROS. Collectively, these results broaden the roles of ROS to encompass microbial differentiation responses and stress responses controlled by MAPK pathways.

## 1. Introduction

NADPH oxidases are a class of enzymes that consist of six to seven transmembrane helices that coordinate two non-identical heme b molecules via four highly conserved histidines. These proteins catalyze the transfer of electrons from NADPH to FADH, from FADH to the two heme b groups and finally to O_2_ leading to the production of superoxide (O_2_^−^) [1]. The first NADPH oxidase was identified in humans because a lack of this enzyme’s activity is the main driver of chronic granulomatous disease that leads to death during early childhood, which has been attributed to a multitude of infections. Leukocytes of these patients have a defect in the enzyme Nox2 and, thus, lack the ability to perform a respiratory burst that is an important parameter of the innate immune defense [2,3,4]. Therefore, it was assumed for some time that NADPH oxidases are restricted to multicellular organisms. Contradicting this assumption, we were able to demonstrate that a NADPH oxidase-like enzyme (Yno1p) exists in the single-celled yeast *Saccharomyces cerevisiae* [5]. Confirming our result, homologous NADPH oxidases have been identified in other mono-cellular ascomycetes [6] and even bacteria [7].

In *S. cerevisiae,* the reactive oxygen species (ROS) produced by Yno1p primarily act as signaling molecules [5]. Although superoxide is directly produced, the main bioactive molecule is hydrogen peroxide [8]. The Yno1p-derived ROS are instantaneously dismutated to H_2_O_2_ by Sod1p [8]. In this way, a ROS producing and an antioxidative enzyme are in a strict dependence of each other to fulfil their function. Due to its localization in the peripheral endoplasmic reticulum (ER), Yno1p engages in signaling with proteins in the plasma membrane. The plasma membrane is an important mediator between the exterior and interior of the cell and is thus an important signaling platform for the cell. A multitude of signaling proteins function at the plasma membrane that include receptors, G-proteins, and protein and lipid kinases. A typical example are the yeast casein kinases, Yck1p and Yck2p, which are tethered to the cytoplasmic side of the plasma membrane by the lipid modification, palmitoylation [9]. Both kinases are involved in sensing extracellular glucose and control mediating its import, but are also involved in bud morphogenesis, pheromone signaling, cytokinesis and endocytosis [10,11,12]. It was shown that hydrogen peroxide derived from the Yno1p–Sod1p interaction stabilizes Yck1p in the plasma membrane. As a direct consequence, mitochondrial respiration is downregulated, and at the same time, glycolysis is promoted [8]. Another prominent example is the evolutionary highly conserved monomeric G-protein Ras2p [13]. Similar to Yck1p/Yck2p, Ras2p is involved in extracellular glucose sensing and can function in modulating mitochondrial respiration [14]. Defects in the mitochondrial electron transport chain can induce translocation of Ras2p from the plasma membrane to the mitochondria to mediate a burst of ROS production by Yno1p [15]. Localized ROS in proximity to the plasma membrane can modulate diverse processes ranging from apoptosis to F-actin polymerization [5]. The pathways that lead to the latter process are the focus of the present manuscript.

Many fungal species are capable of undergoing a nutrient-dependent growth response commonly referred to as filamentous growth. Filamentous occurs in many fungal species, and in fungal pathogens can be required for virulence [16]. In the major human fungal pathogen *Candida albicans*, the NADPH oxidase Fre8p resides in the plasma membrane, particularly at the growing tip of the cell. As in *S. cerevisiae*, the NADPH oxidase fulfills its function in concert with a superoxide dismutase. Unlike in baker´s yeast, the resulting hydrogen peroxide acts in an extracellular manner. The resulting ROS burst is important for the transition from yeast to hyphal growth and differentiation to the infectious form of *C. albicans.* In a mouse model of candidiasis, ∆*fre8*/∆*fre8* strains showed significantly shortened hyphae [6].

**Figure 1:**
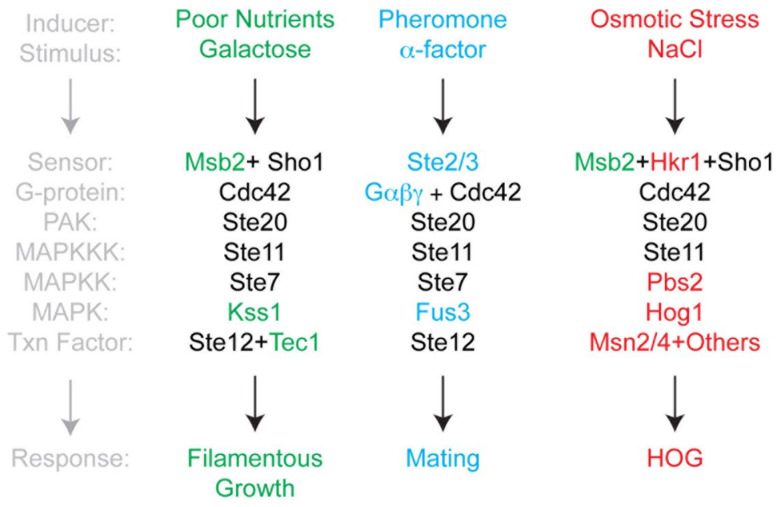
Cdc42-dependent MAPK pathways in yeast. Three yeast MAPK pathways share components, yet induce different responses to different stimuli. Pathway components common to multiple pathways are shown in black. Pathway-specific proteins are colored (red letters: hyperosmolarity glycerol response (HOG) pathway; blue letters: pheromone response; green letters: filamentous growth). Not all components are shown.

In contrast to *C. albicans*, *S. cerevisiae* is not capable of forming true hyphae, but rather forms pseudohyphae, where cells fully separate by cytokinesis [17,18]. During pseudohyphal growth, cells become elongated due to an extended period of apical growth due to a delay in the cell cycle, which involves vesicle trafficking along a highly polarized actin-based cytoskeleton [19,20]. Cells also reorganize their polarity to grow outward from their ends at the distal poles [21]. In addition, cells remain attached to each other (and can attach to surfaces) due to contacts mediated by cell adhesion molecules. One tractable phenotype in *S. cerevisiae* and other fungal species is invasive growth, where filamentous cells penetrate into surfaces [22]. The route to filamentous growth in *S. cerevisiae* is complex and integrates signals from different pathways including the Ras2p-cAMP-protein kinase A (PKA) pathway [18,23,24,25], the Rim101 pathway [26], the Snf1p Nrg1p/Nrg2p pathway and a mitogen activated protein (MAP) kinase pathway commonly referred to as the filamentous growth or fMAPK (filamentous growth MAPK) pathway (Figure 1, [27]). The fMAPK pathway is regulated by a cell-surface glycoprotein, Msb2p, which controls the activity of the Rho GTPase Cdc42p [28]. Like Ras2p, Cdc42p is a monomeric GTPase of the RAS superfamily. When activated by GTP binding, Cdc42p interacts with the serine/threonine p21 activated kinase (PAK) Ste20p, which controls a MAP kinase cascade consisting of Ste11p (MAPKKK), Ste7p (MAPKK), and Kss1p (MAP kinase) (Figure 1) [29]. Kss1p phosphorylates and activates the transcription factors Ste12p and Tec1p, which bind to so called “Filamentation and invasive Response Elements”. These consensus sequences are found in the promoter regions of several genes that are necessary for filamentous growth [27,30]. Many of the pathways that regulate filamentous growth can influence each other’s activities. For example, Ras2p regulates the activity of the fMAPK pathway [31].

The MAP kinase pathway that regulates filamentous growth is composed of proteins that can also function in other MAP kinase pathways in the same cell (Figure 1). Many of the proteins, including Cdc42p, Ste20p, Ste11p, and Ste7p also regulate the mating or pheromone response pathway [32]. The mating pathway is required for haploid cells of complementary cell types to form diploids. During mating, cells express peptide pheromones (**a**- and alpha-factor) that are recognized by G-protein coupled receptors (GPCR, Ste2p, and Ste3p) on complementary cell types. Detection of pheromone by receptor leads to transcriptional induction of target genes, cell cycle arrest, and morphogenetic changes that prepare the cell to interact with its mating partner. Fusion of the two cell types leads to the formation of a diploid cell. One response of yeast cells that come into contact with pheromones is a morphological change known as shmoo. The formation of shmoos is mainly driven by the actin cytoskeleton: actin patches are concentrated in the shmoo tip, whereas actin cables can be found alongside the axis of polarization [33]. This is a further connecting link to filamentous growth in which primarily actin patches play an important part and are mostly polarized to the distal tip of the outwardly growing cell [20]. It has previously been shown that addition of non-physiological concentrations of alpha factor induce a burst of ROS production after 1.5 h accompanied with apoptotic cell death after 3.5 h. Under native conditions (mixing **a** with alpha cells) this phenomenon was observed at reduced levels (~30% of all cells) [34].

In addition to mating and filamentous growth, Cdc42p, Ste20p, and Ste11p proteins also regulate one of two redundant branches of the p38-type hyperosmolarity glycerol response (HOG) pathway, which mediates the tolerance to osmotic stress (Figure 1) [35]. In the HOG pathway, two mucin-type glycoproteins function as sensors, one being Msb2p, which also functions in the fMAPK pathway and the other, Hk1p, which does not [36,37,38,39]. These proteins along with the adaptor Sho1p regulate Cdc42p, Ste20p, and Ste11p, which in turn regulates the MAPKK Pbs2p to control the activity of the MAP kinase Hog1p. Hog1p regulates at least five transcription factors: Msn2p, Msn4p, Hot1p, Sko1, and Smp1p [40]. A potential target of Msn2p is *YNO1*, which suggests that Yno1p might play a role in the osmotic stress response [41]. Therefore, signaling pathways can share components yet induce different responses, which can be critical for the response to stress and cell differentiation. Among the responses to osmotic stress are fragmentation of the vacuole and disruption of the actin cytoskeleton (for a detailed review see [40]). Vacuolar morphology is highly dynamic, and the underlying fission and fusion events are dependent on actin polymerization, which is also mediated by Cdc42p [42]. During vegetative growth, two to three vacuoles exist in yeast cells at the same time. Hypotonic conditions initiate vacuole fusion leading to a large vacuole, whereas salt stress initiates vacuole fission resulting in vacuole fragmentation [43].

In the present study, we explore how Yno1p might impact signaling functions during osmotic stress, pheromone response, and filamentous/invasive growth. We show that Yno1p impacts aspects of these processes in distinct ways. One way in which Yno1p might impact these processes is by its ability to regulate the actin cytoskeleton. Because morphogenetic processes and signaling events require a proper functioning actin cytoskeleton, Yno1p may integrate ROS signals by this mechanism. Therefore, ROS generation may be an important part of the cellular response to diverse stimuli.

## 2. Material and Methods

### 2.1. Strains and Media

All strains used in the study are either based on *S. cerevisiae* strains BY4741 (*MATa his3*Δ*1 leu2*Δ*0 met15*Δ*0 ura3*Δ*0*), BY4742 (*MAT*α *his3*Δ*1 leu2*Δ*0 lys2*Δ*0 ura3*Δ*0*) [44], or Σ1278b (*MAT*α; *ura3-52*; *trp1∆::hisG*; *leu2∆::hisG*; *his3∆::hisG*) [45,46]. For details on strains used in this study, their construction, as well as media composition in which the strains were grown, see Supplementary Material and Methods.

### 2.2. Cloning Experiments

The expression vector p416GPD was used in the study. The *LAS17* open reading frame was amplified by PCR from genomic yeast DNA by using Phusion^®^ High-Fidelity DNA Polymerase (NEB, Ipswich, MA, USA) using the following primers: *LAS17* forward (5′-CGG GAT CCA TGG GAC TCC TAA ACT C-3′) and *LAS17* reverse (5′-CCG CTC GAG TCA CTT ATC GTC ATC CTT GTA ATC CCA ATC ACC ATT GT-3′). The resulting PCR product was cloned into the vector using BamHI and XhoI (NEB, Ipswich, MA, USA). Successful cloning was confirmed by sequencing at Eurofins-MWG-OPERON.

### 2.3. DHE Measurements

The dihydroethidium (DHE) assay was performed as described in [47]. The yeast strains were grown for 16h in liquid SC−glucose. Cells were collected by centrifugation (5000 rpm 2′) and washed twice with 1 × PBS (phosphate buffered saline). Approximately 5 × 10^6^ cells were resuspended in 180 µL 1 × PBS containing 5 µg/mL DHE (Sigma-Aldrich/Merck, Darmstadt, Germany) and incubated for 15 min in the dark. DHE fluorescence was determined using an Anthos Zenyth 3100 plate reader (excitation: 485 nm; emission: 595 nm). Cell numbers were determined with an electric field multichannel cell counting system (CASY^TM^; Schärfe; Germany).

### 2.4. Reporter Measurements

Cells were washed twice in 1 × PBS and 5 × 10^6^ cells were resuspended in 200 µL 1 × PBS. GFP (green fluorescence protein) fluorescence was determined using an Anthos Zenyth 3100 plate reader (excitation: 485 nm; emission: 535 nm) as described [48]. The autofluorescence (cells without GFP expression) was subtracted from the signal reported. For experiments with wiskostatin (racemate) (Calbiochem/Sigma-Aldrich, Merck, Darmstadt, Germany), cells were grown for 18 h in liquid SC (synthetic complete) medium; 200 µM wiskostatin was added for 3–4 h. For experiments with isoamyl alcohol (IAA), cells were diluted to an OD_600_ (optical density at a wavelenght of 600 nm)= 0.1 in liquid YEPD (yeast extract peptone dextrose) medium. Moreover, IAA (0.5%) was added, and cells were grown for 36–48 h. To evaluate osmotic stress, cells were diluted to an OD_600_ = 0.1 in liquid YEPD medium. A total of 1 M NaCl was added and cells were grown for 36–48 h. To evaluate filamentous growth, a preculture was grown in SC-raffinose (3%). Cells were diluted to an OD_600_ = 0.1 in SC-galactose (3%) and grown for~36 h.

### 2.5. Spot Test Assay

Yeast strains (control; single and double gene deletions) were grown in liquid SC-glucose medium at 24 °C until stationary phase. Cells were diluted to OD_600_ values of 3.0, 1.0, 0.3, and 0.1. SC-glucose semi-solid agar medium was prepared containing wiskostatin in a range from 10 to 20 μM. To semi-solid agar media, 10 µL aliquots of the diluted yeast strains were spotted. Plates were incubated for 3–4 days at 24 °C.

### 2.6. Assays to Evaluate Filamentous/Invasive Growth

The plate-washing assay was performed as described [22]. The single-cell invasive growth assay was performed as described [49]. Differential interference contrast (DIC) microscopy was performed with an Axioplan 2 fluorescent microscope (Zeiss, Oberkochen, Germany) with a Plan-Apochromat 100X/1.4 (oil) objective (N.A. 1.4) (cover slip 0.17) (Zeiss, Oberkochen, Germany). Cells were examined at multiple focal planes with an Axiocam MRm camera (Zeiss, Oberkochen, Germany) and AxioVision 4.4 software (Zeiss, Oberkochen, Germany). Adjustments to contrast and brightness were performed using Adobe Photoshop (Adobe, San Jose, CA, USA). ImageJ analysis (http://rsb.info.nih.gov/ij/) was used to quantitate invasive growth [50]. Background intensity was determined for each spot and subtracted from the densitometry of the area of invaded cells. Invasive growth assays were performed in biological replicates (N > 3). The variation in between samples was typically less than 15% across trials, as assessed by independent quantitation methods, ImageJ and OpenLab Software.

### 2.7. Immunoblot Analysis

Immunoblots were performed as described [51]. Proteins were prepared from cell extracts from cells grown under the indicated conditions and analyzed by sodium-dodecyl-sulfate (SDS) polyacrylamide gel electrophoresis (PAGE), using 10% acrylamide in the running gels. Proteins were transferred from gels to nitrocellulose membranes that were probed with the indicated antibodies. Antibodies that recognize GFP (#11814460001, clones 7.1 and 13.1, Roche) were used at 1:1000 dilution. Antibodies that recognize Pgk1p (Life Technologies; Camarillo, CA, USA; Cat #459250) were used at 1:10,000 dilution. Secondary antibodies goat anti-mouse IgG–HRP (Bio-Rad Laboratories, Hercules, CA, USA; Cat #170-6516) were used for detection of primary antibodies. Band intensity was measured by Image Lab Software (Bio-Rad, Hercules, USA) using unsaturated exposures. Background subtraction was performed based on the software user guidelines. Band intensities were compared to total protein levels based on Pgk1p levels.

### 2.8. Osmotic Stress and FM4-64 Staining

FM4-64 staining with small modifications was performed as described [52]. Yeast cells were diluted to an OD_600_ = 0.1 and were grown for 12 h in liquid SC-medium. An aliquot (2 mL) was washed once in YEPD and resuspended in 100 µL YEPD supplemented with 20 µM FM4-64. After a 20 min incubation at 28 °C, cells were wash with SC-medium and incubated for 1 h in 100 µL liquid SC medium. Moreover, 50 µL of this cell suspension was used as a control, 50 µL was supplemented with 1 M NaCl and incubated for >30 min. Cells stained by FM4-64 were analyzed with a × 100 objective (NA = 1.4) using either a Carl Zeiss AG Axioscope (Oberkochen, Germany) or a Nikon (Tokyo, Japan) Eclipse Ni-U equipped with a DS-Fi2 digital camera.

### 2.9. Pheromone Response Assay

Cells were grown to late exponential phase in liquid SC medium. Shmooing and apoptotic cell death was induced by the addition of 100 µg/mL alpha-factor (GenScript, Piscataway, NJ, USA). After incubation for 4 h at 28 °C under constant shaking, the appearance of shmoos was evaluated by light microscopy. Cell survival was calculated by plating 500 cells on YEPD medium and counting the number of colonies after 3 days of incubation at 28 °C.

### 2.10. Actin Morphology

Cells were cultivated in 3 mL of SC-Ura media in 6-well plates. The cell suspension was then mixed with fresh SC-Ura medium (1:1) and galactose up to 3% was added after 1 h precultivation. The cells were inspected in the cultivation medium, mounted on a glass slide, covered with a coverslip, and directly imaged at room temperature. Only the first three snapshots of each sample were used for analysis. The distribution of TagRFP-T was analyzed with a 100x Plan Apochromat objective (NA = 1.4). An Olympus IX-81 inverted microscope (Tokyo, Japan) equipped with a Hamamatsu Orca/ER digital camera and the Olympus Cell RTM detection and analyzing system was used. TagRFP-T fusion was detected using RFP filter block (U-MWIY2, excitation; 545–580 nm; emission 610 nm). Images were processed and merged using Olympus Cell RTM (xcellence RT) and Adobe CS6 software. Images presented as Z-stack are maximal projections of stacks obtained using a 0.4-μm steps. Images presented as T-stacks are minimum intensity projection of fluorescence from 15 captured frames. Deconvolution of images obtained by Z-sectioning was done by Wiener filter (WF). Time lapse settings: The image acquisition parameters of T-stack in Figure 9 were one frame per 5 s (fifteen frames in total), and in supplementary movies, one frame per 5s (fifty frames in total).

### 2.11. Statistical Analysis

Data are presented as arithmetic means ± S.D. We used a one-way ANOVA test including Tukey HSD (honestly significant difference) and a student’s t-test for statistical analysis. Results with a *p* < 0.05 were considered to be statistically significant.

### 2.12. Gene Accession Numbers

*YNO1*: S000003128; *HOT1*: S000004783; *KSS1*: S000003272; *RAS2*: S000005042; *ARP2*: S000002187; *ARP3*: S000003826; *ABP140*: S000005765; *CDC42*: S000004219; *PBS2*: S000003664; *STE7*: S000002318; *BEM2*: S000000957; *TEC1*: S000000287; *RIM101*: S000001019; *STE11*: S000004354; *SMP1*: S000000386; *STE12*: S000001126; *SNF1*: S000002885; *LAS17*: S000005707; *NRG2*: S000000270; *NRG*1: S000002450; *MSN2*: S000004640; *SOD1*: S000003865; *HOG1*: S000004103; *MSB2*: S000003246; *VRP1*: S000004329; *YCK1*: S000001177; *YCK2*: S000005098; *STE20*: S000000999; *SKO1*: S000005111

## 3. Results

### 3.1. Establishment of an YNO1 Expression Reporter

In a previous study [5], we have shown that the ROS produced by the NADPH oxidase Yno1p at physiological levels are not harmful for the cell, but act primarily as signaling molecules. Moreover, overproduction of the Yno1p protein, which induces a burst of ROS, resulted in the induction of apoptotic cell death [5]. To gain further insight into the pathways that may be regulated by Yno1p, an expression reporter was constructed. The *YNO1* ORF was replaced by GFP; thus, placing the fluorescent protein under control of the native *YNO1* promoter (P*_YNO1_* ∆*yno1*::GFP-*HIS3*). The necessary genetic manipulations are summarized in the Supplementary Material and Methods section, and the resulting genetic alterations on chromosome VII are shown in Supplementary Figure S1.

**Figure 2:**
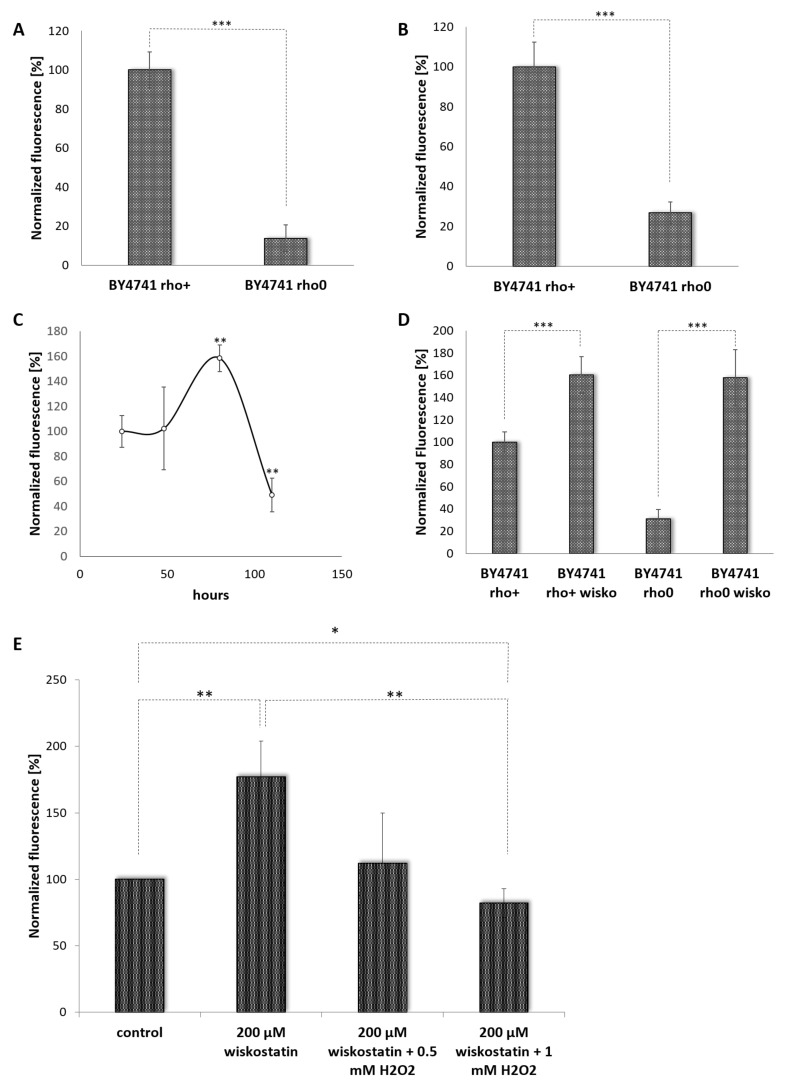
A GFP-based *YNO1*-reporter. (**A**) Superoxide levels were measured by using the fluorescent probe DHE. The wild type strain BY4741 (rho^+^) and the *petite* strain BY4741 (rho0) were analyzed (N = 4–7). In (**B**–**E**), the expression of *YNO1* was studied by measuring GFP fluorescence. A GFP-*HIS3* cassette was used to replace the native *YNO1* ORF. In (**B**), the strains BY4741 ∆yno1::GFP-*HIS3* rho+ and BY4741 ∆yno1::GFP-*HIS3* rho0 were compared (N = 6). In (**C**), the expression of *YNO1* was analyzed at the transition from late exponential phase to early stationary and stationary phase in the strain BY4741 ∆yno1::GFP-*HIS3* (N = 4). (**D**) shows the expression of *YNO1* in the strains BY4741 ∆yno1::GFP-*HIS3* rho+ and BY4741 ∆yno1::GFP-*HIS3* rho0 after addition of 200 µM wiskostatin (wisko, N = 4). In (**E**), wiskostatin (200 µM)-induced expression of *YNO1* is reversed by addition of 0.5 mM or 1 mM H_2_O_2_ (N = 4) (*: *p* < 0.1; **: *p* < 0.05; ***: *p* < 0.01).

The *YNO1* expression reporter was tested under three well-defined settings. One setting was cells lacking mtDNA. It is well established that a rho0 *petite* yeast strain (a strain completely devoid of mtDNA) shows reduced ROS production [53,54,55]. By using a dihydroethidium (DHE) assay, ROS levels were compared between the rho0 strain and the corresponding wild type strain. DHE was initially used as a superoxide specific probe, but recently it was shown that this dye can be oxidized by hydrogen peroxide in the presence of heme proteins [56]. By this test, the rho0 strain showed reduced levels of ROS production (Figure 2A). The DHE fluorescence signal in the *petite* strain was more than 80% decreased. Currently, it is assumed that complex III of the respiratory chain is responsible for 80% of the cellular superoxide levels in yeast cells [54]. Surprisingly, the ROS levels in *petite* strains correlated well with the expression of Yno1p. The wild type strain showed a 5-fold higher expression of P*_YNO1_* ∆*yno1*::GFP-*HIS3* than the non-respiring strain (Figure 2B). This result indicates that cross talk occurs between the mitochondria and the expression of the gene encoding NADPH oxidase. This phenomenon of ROS-induced ROS has been described in other model systems [57,58]. Previously, we and others have demonstrated that Yno1p contributes to~20% of the cellular superoxide levels under normal growth conditions [5,8,15,59]. As shown below, an increase in Yno1p expression accompanied by an increase in ROS production is needed under well-defined cellular settings.

The second condition was in cells experiencing ER stress. It has been established that Yno1p protein levels are controlled by the endoplasmic reticulum-associated degradation (ERAD) pathway. After a diauxic shift, Yno1p levels were absent at the ER [15]. By the Yno1p reporter system, we found that the expression of *YNO1* was quite stable during exponential growth and leads to a burst of expression in early stationary phase (~70 h of growth). After 100 h, the GFP signal significantly decreases (Figure 2C). This discrepancy to the data presented in Leadsham at al. [15] could be explained by the fact that the Yno1p itself may be specifically turned over by the ERAD system, whereas GFP is quite stable. GFP may therefore be a useful reporter for switch-on (inducing), but not switch-off (repressing) conditions [60].

The third condition was experiments interrogating the actin cytoskeleton. We have previously shown that a ∆*yno1* strain is sensitive to the addition of wiskostatin [5]. Wiskostatin was shown to interact with the evolutionary highly conserved protein N-WASp (isolated from *Xenopus laevis* eggs) thus interfering with F-actin polymerization [61]. Treating cells harboring the Yno1p reporter with wiskostatin led to an increase in GFP signal. Several wiskostatin concentrations were tested in a range from 50–300 µm wiskostatin and a peak was reached at a concentration of 150–200 µM with a >60% increase in GFP fluorescence. In a *petite* strain, the difference was even more striking, which showed an~8-fold increase in the expression of the P*_YNO1_* ∆*yno1*::GFP-*HIS3* construct (Figure 2D). Under the described conditions, addition of either 0.5 or 1 mM H_2_O_2_ reduced the expression of GFP to the same levels as seen in control cells (Figure 2E).

**Figure 3:**
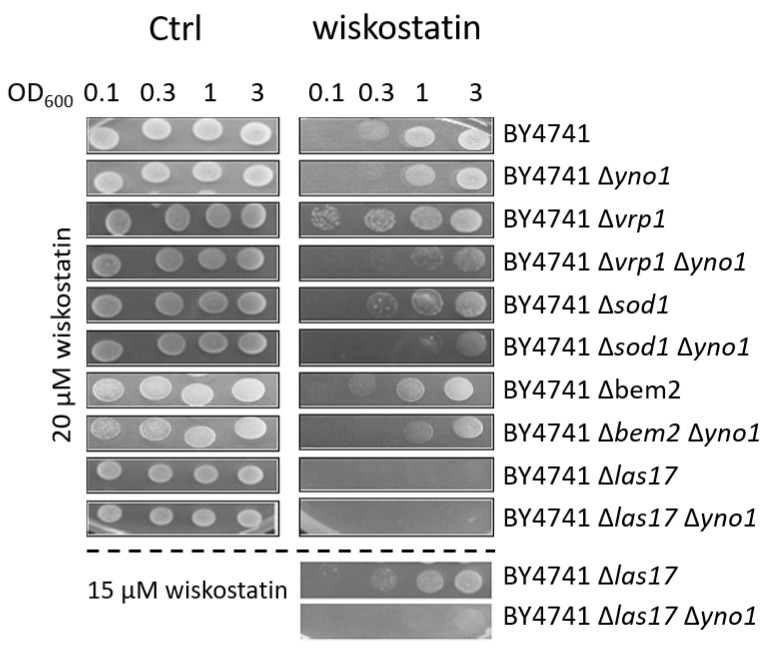
The sensitivity of mutants to wiskostatin by spot test analysis. Serial dilutions (OD_600_ of 3, 1, 0.3, and 0.1) of the strains BY4741, BY4741 ∆*yno1*, BY4741 ∆*vrp1*, BY4741 ∆*sod1*, BY4741 ∆*bem2*, BY4741 ∆*las17*, BY4741 ∆*yno1* ∆*vrp1*, BY4741 ∆*yno1* ∆*sod1*, BY4741 ∆*yno1* *∆bem2*, and BY4741 ∆*yno1* ∆*las17* were spotted on SC-glucose plates, SC-glucose +20 µM wiskostatin plates and SC-glucose +15 µM wiskostatin plates. At least three biological replicates were tested on plates containing concentrations of wiskostatin in a range from 0–100 µM. Representative images and concentrations were chosen.

To define how Yno1p might impact polymerization of F-actin, a spot test was performed. Serial dilutions of yeast cells were spotted on SC plates containing 20 µM wiskostatin and incubated for 3–4 days at 24 °C, which was permissive for growth for the temperature-sensitive strains tested. Under the conditions examined, the ∆*las17* and ∆*vrp1* strains showed slow growth at 28 °C, and the ∆*las17* ∆*vrp1* double deletion strain was not viable. Confirming previous results, deletion of *YNO1* also led to sensitivity to wiskostatin. The yeast homologue of N-WASp is Las17p, which together with the Arp2/3 complex, promotes the nucleation of branched actin filaments [59]. Surprisingly, deletion of the proposed target of wiskostatin, Las17p, made the cells hypersensitive to the addition of 20 µM of wiskostatin (Figure 3). Therefore, it can be excluded that the N-WASp inhibitor solely binds to Las17p in yeast cells and may indicate that there is at least one other interacting partner. A likely candidate for the binding of wiskostatin is Vrp1p (verprolin; very proline-rich). Vrp1p binds to the N-terminal region of Las17p, which is also rich in proline, and together the two proteins activate the Arp2p/3p complex [62]. Both Vrp1p and Las17p promote the nucleation of F-actin independently of the Arp2/3 complex [63]. In fact, wiskostatin had only a modest effect on growth of the ∆*vrp1* strain. A ∆*yno1* ∆*vrp1* double deletion strongly amplified the wiskostatin sensitivity compared to the corresponding single deletions. By reducing the wiskostatin concentration, the ∆*las17* showed growth compared to the ∆*las17* ∆*yno1* double deletion strain.

Directly upstream in the pathway that regulates Las17p is the Rho GTPase Cdc42p. This GTPase is essential, therefore a ∆*bem2* strain was analyzed. Bem2p is the GAP of Cdc42p and upon its deletion the activity of Cdc42p is disturbed. A double deletion strain of *BEM2* and *YNO1* was hyper-sensitive to wiskostatin and showed a slower growth on plates containing wiskostatin than the appropriate single gene deletions. Yno1p itself is not capable of producing the signaling molecule hydrogen peroxide and is therefore most probably dependent on the superoxide dismutase Sod1p [8]. This assumption was tested by using a *∆yno1 ∆sod1* double deletion strain that showed a high sensitivity to wiskostatin. If any, the phenotype of a *SOD1* single gene deletion was modest. A further downstream target of Cdc42p is the PAK Ste20p that mediates osmotic stress response [64], pheromone response [65] and filamentous growth [27]. These three pathways will be studied below.

### 3.2. Yno1p and the Osmotic Stress Response

**Figure 4:**
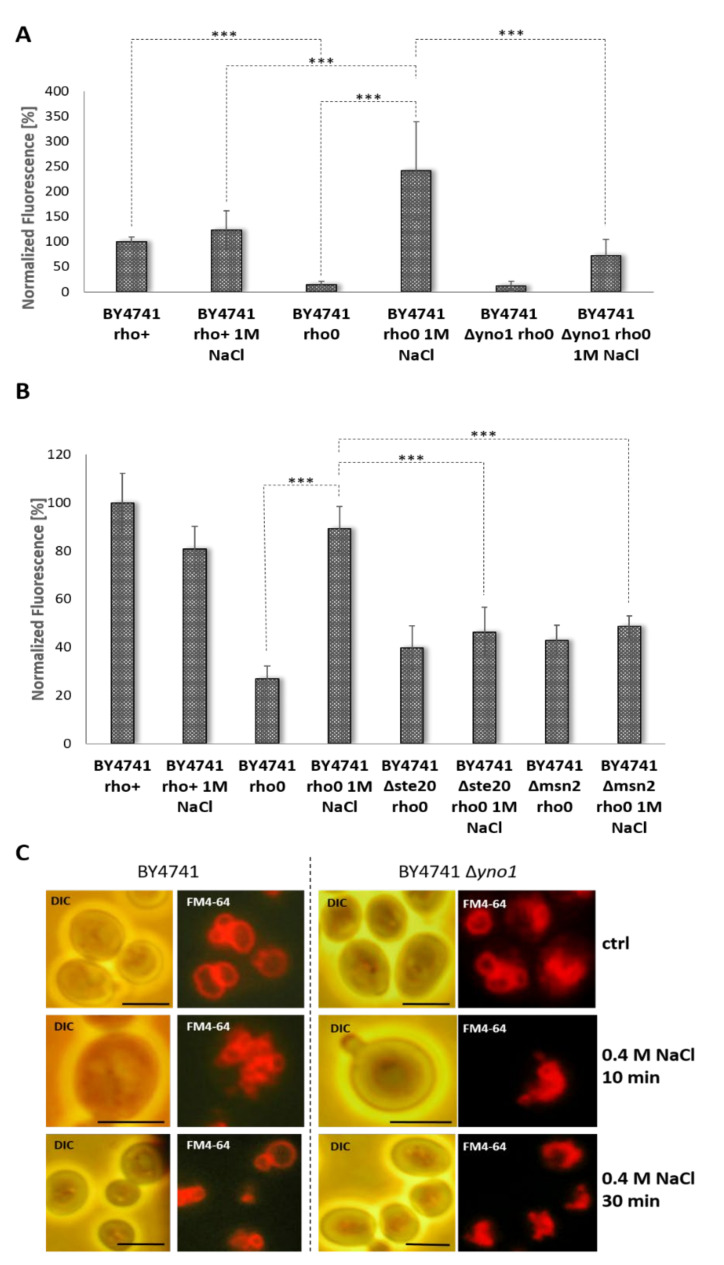
*YNO1* reporter levels and vacuolar morphology in response to osmotic stress. (**A**) Superoxide levels were measured using the dye DHE in the strains BY4741 rho+, BY4741 rho0, and BY4741 ∆*yno1* rho0 (N = 4–7). In (**B**) the *YNO1* expression in the strains BY4741 rho+, BY4741 rho0, BY4741 ∆*ste20* rho0 and BY4741 ∆*msn2* rho0 was measured using a GFP-reporter (N = 4–6). In (**A**) and (**B**), osmotic stress response was induced by the addition of 1 M NaCl (***: *p* < 0.01). In (**C**), vacuolar membranes were stained with the lipophilic dye FM4-64. Osmotic stress was induced by addition of 0.4 M NaCl. Scale bars represent 5 µm. The assay was performed in triplicate, and representative images are shown.

It has been reported that salt stress interferes with the cell cycle, leading to growth arrest and ultimately the apoptosis of yeast cells [66]. In subsequent studies, it was shown that in this case programmed cell death is not accompanied by increased ROS levels. Such elevated ROS levels were only observed in specific deletion mutants (e.g., a Δ*sro7* strain) [67]. Our analysis confirmed this finding. Addition of 1 M NaCl did not lead to a significant increase in superoxide levels in the BY4741 background (Figure 4A). In contrast to the situation in the wild type strain, the absence of mitochondrial DNA and, thus, mitochondrial respiration led to a high response to the addition of NaCl.

The addition of 1 M NaCl increased the amount of ROS in the strain BY4741 rho0 by >17-fold. Under these conditions, the amount of ROS in the *petite* strain surpassed superoxide levels in untreated wild type cells. The increase of ROS upon NaCl treatment was largely dependent on the yeast NADPH oxidase. Deletion of *YNO1* in the *petite* background led to a strong reduction in ROS levels, although a non-statistical significant increase was still observed. These findings were also mimicked by the *YNO1* reporter expression: no increase in P*_YNO1_* ∆*yno1*::GFP-*HIS3* expression was observed in the rho^+^ background, whereas a 3-fold expression was found in the rho0 background upon addition of 1 M NaCl (Figure 4B). In a *petite* strain also lower concentrations of NaCl (0.5 mM and 0.7 mM) induced an expression of Yno1p albeit on reduced levels.

We next sought to test whether the osmotic stress induced Yno1p expression is dependent on the Cdc42p/Ste20p proteins. Cdc42p and its effector Ste20p regulate one branch of the HOG pathway (Figure 1) [40,64]. Interestingly, in cells lacking Ste20p, the increase in GFP-signal by NaCl did not occur. Another protein that is important in coping with osmotic stress is Msn2p. This is a cytosol-localized transcription factor that upon stress induction translocates to the nucleus and mediates the expression of stress response genes [68,69]. It is predicted that this transcription factor also binds to the promoter region of *YNO1* [41,70]. During osmotic stress, Msn2p migrates to the nucleus (after~8 min) shuttles back to the cytosol presumably once cellular adaptation occurs (after 15 min) [71]. Deletion of *MSN2* also abolished the increase in *YNO1* expression during salt stress (Figure 4B). Another predictor for the change in osmolarity is vacuolar morphology. In the BY4741 background, these changes in vacuolar morphology are transient [52]. To assay mitochondrial morphology BY4741 and the BY4741 ∆*yno1* mutant, cells were prestained with the dye, FM4-64 [72]. FM4-64 is a lipophilic dye that intercalates into the plasma membrane, and after internalization by endocytic events, stains the vacuolar membrane and other internal compartments. In most untreated control cells (Figure 4C), three distinct vacuoles were observed. The addition of 0.4 M NaCl induced fragmentation of vacuoles after 10 min. By 20 min and until cells adapted to stress, the fragmented vacuoles underwent fusion. In cells lacking *YNO1*, untreated cells showed a higher fragmentation of vacuoles, although on a very subtle level. Similar to wild type cells, addition of NaCl resulted in a complete fragmentation of vacuoles. After 30 min of incubation, no sign of adaptation was observed in the ∆*yno1* background, and it took another 30 min until the vacuoles started to revert back to the normal phenotype.

### 3.3. Yno1p and the Pheromone Response

**Figure 5:**
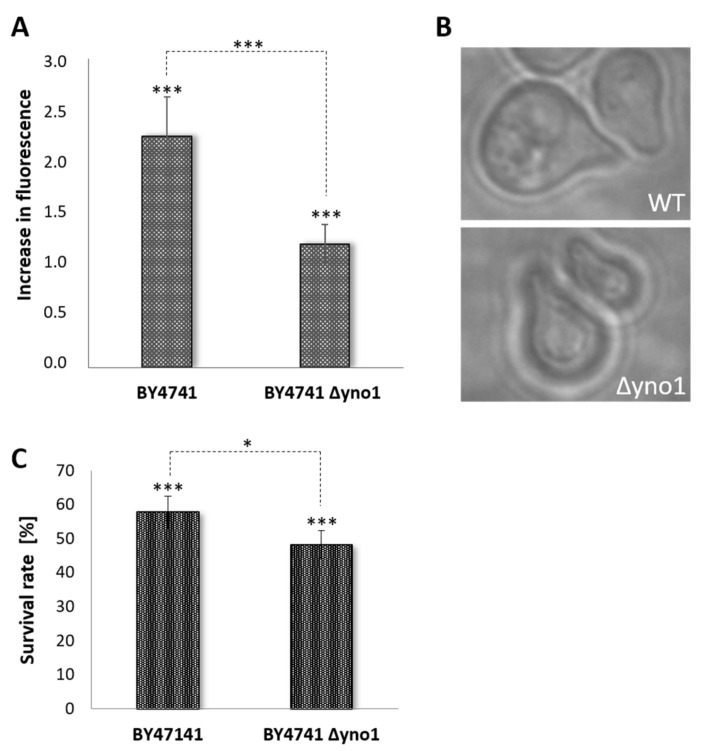
The role of Yno1p in pheromone response. (**A**) DHE fluorescence was analyzed in the strains BY4741 and BY4741 ∆*yno1* in untreated controls as well as after addition of alpha factor (100 µg/mL) (N = 8). In (**B**), differential interference contrast microscopy images of shmoos in the BY4741 and BY4741 ∆*yno1* background are shown after addition of alpha factor (100 µg/mL). In (**C**), the survival rate in the strains BY4741 and BY4741 ∆*yno1* was calculated by survival plating. Cell death was induced by addition of alpha factor (100 µg/mL). In untreated cells (BY4741 and BY4741 ∆*yno1*), no difference in survival was observed. For (**A**,**C**): each bar represents the comparison between untreated and treated yeast strains (either BY4741 or BY4741 ∆*yno1*), asterisks above the bars indicate statistical significance between with/without treatment, asterisks above the brackets represent statistical significance between the different yeast strains (BY4741 and BY4741 ∆*yno1*) upon treatment with alpha-factor (*: *p* < 0.1; ***: *p* < 0.01).

The addition of non-physiological concentrations of alpha-factor results in a burst of ROS production [34]. In fact, we found that most of the ROS produced in this setting was dependent on the yeast NADPH oxidase. The wild type strain BY4741 showed a >2-fold increase in DHE fluorescence after addition of 100 µg/mL alpha factor. In the *∆yno1* strain a lower increase in ROS was observed (Figure 5A).

Surprisingly, the formation of shmoos (Figure 5B) was not affected by a deletion of *YNO1*. Although the ROS level decreased in the ∆yno1 deletion background, the survival rate was not increased, but on the contrary slightly decreased when compared to the wild type (Figure 5C). Confirming the results of Severin and Hyman [34], an overdose of alpha factor initiates a process that kills approximately 50% of cells independent of the genetic background.

### 3.4. Evaluating the Role of Yno1p in Regulating Filamentous Growth

**Figure 6:**
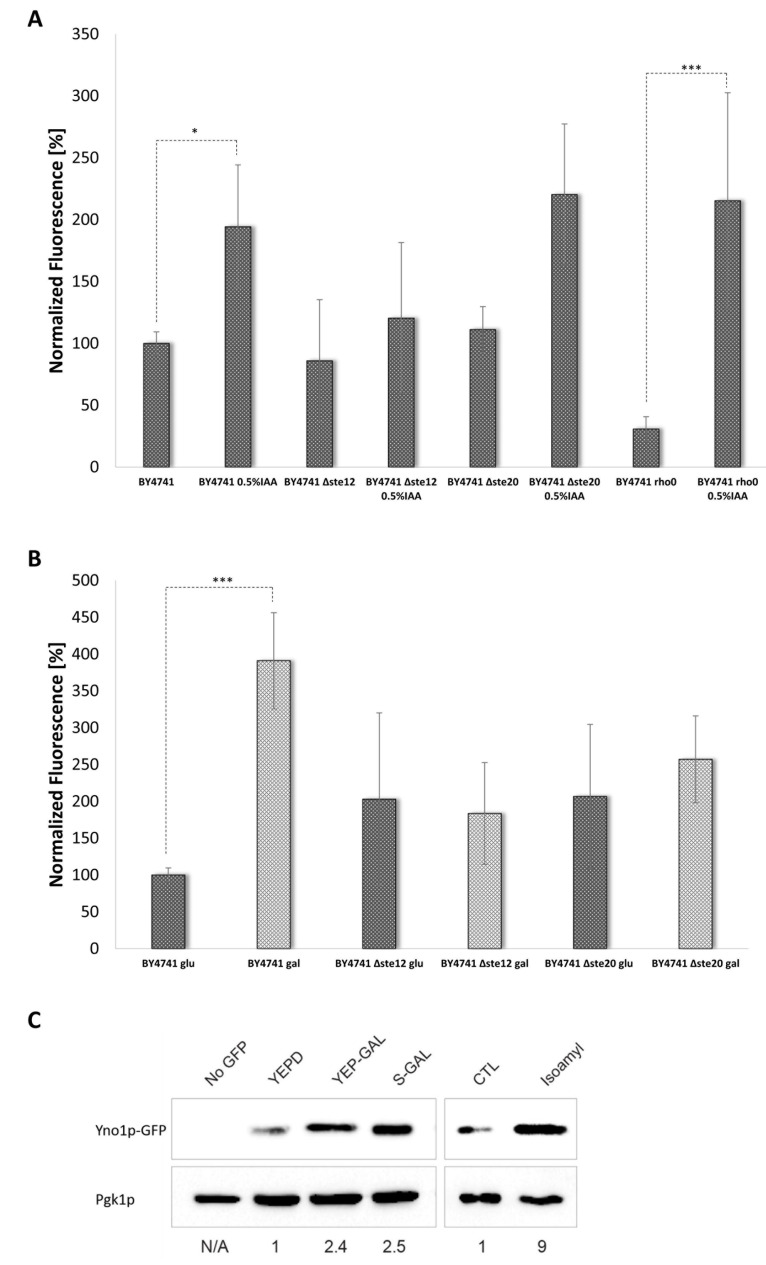
Expression of *YNO1* under conditions that stimulate filamentous growth. The expression of *YNO1* was analyzed using the GFP-reporter construct in the strains BY4741 rho+, BY4741 rho0, BY4741 ∆*ste20,* and BY4741 ∆*ste12.* Filamentous growth was induced by addition of 0.5% isoamyl alcohol (IAA) for 36 h (**A**) or growth in galactose media (**B**) (*: *p* < 0.1; ***: *p* < 0.01). In (**C**), immunoblot analysis of cells harboring a *YNO1-GFP* fusion (BY4741 *YNO1*-GFP::*HIS3*) were grown under the indicated conditions. A control strain (BY4741) was used for the no-GFP control. Equivalently run blots were probed using antibodies to Pgk1 as a loading control. Band intensity of GFP normalized to Pgk1 levels is shown below the blots and was determined by Bio-Rad XRS^+^ Image Lab software.

In addition to regulating the HOG pathway and the response to pheromone, Cdc42p and Ste20p also regulate the filamentous growth pathway [46], which is one of the signaling pathways that induces invasive/filamentous growth in response to nutrient limitation [27]. Filamentous growth in yeast can be induced in several ways. “Fusel” alcohols, such as isoamyl alcohol (IAA), can induce filamentous growth [73,74], as does growth in non-preferred carbon (e.g., galactose) [75] and nitrogen sources [46]. Both 0.5% isoamyl alcohol (IAA) (Figure 6A) and 2% galactose (Figure 6B) induced the Yno1p reporter (of P*_YNO1_* ∆*yno1*::GFP-*HIS3)*. To confirm this result, *YNO1* was genomically tagged with GFP (BY4741 *YNO1-GFP::HIS3*) and analyzed by immunoblot analysis. Galactose or IAA treatment caused an increase in the level of Yno1p-GFP (2.5-fold and 9 fold, respectively) (Figure 6C). Deletion of *STE20* caused a reduction in Yno1p expression in SC-galactose media (Figure 6B). An Yno1p reporter signal was still detectable in the ∆*ste20* background after addition of IAA. A downstream target of the yeast MAP kinase cascade consisting of several serine/threonine kinases (Ste20p → Ste11p → Ste7p → Kss1p) are the transcription factors Tec1p and Ste12p [27]. Similar to Msn2p, Ste12p is predicted to bind to the *YNO1* promoter region [41,76]. Under the induction conditions tested (galactose and IAA), deletion of *STE20* resulted in reduced *YNO1* expression (Figure 6A,B). This result indicates that *YNO1* expression may be regulated by the MAP kinase cascade that regulates filamentous growth.

**Figure 7:**
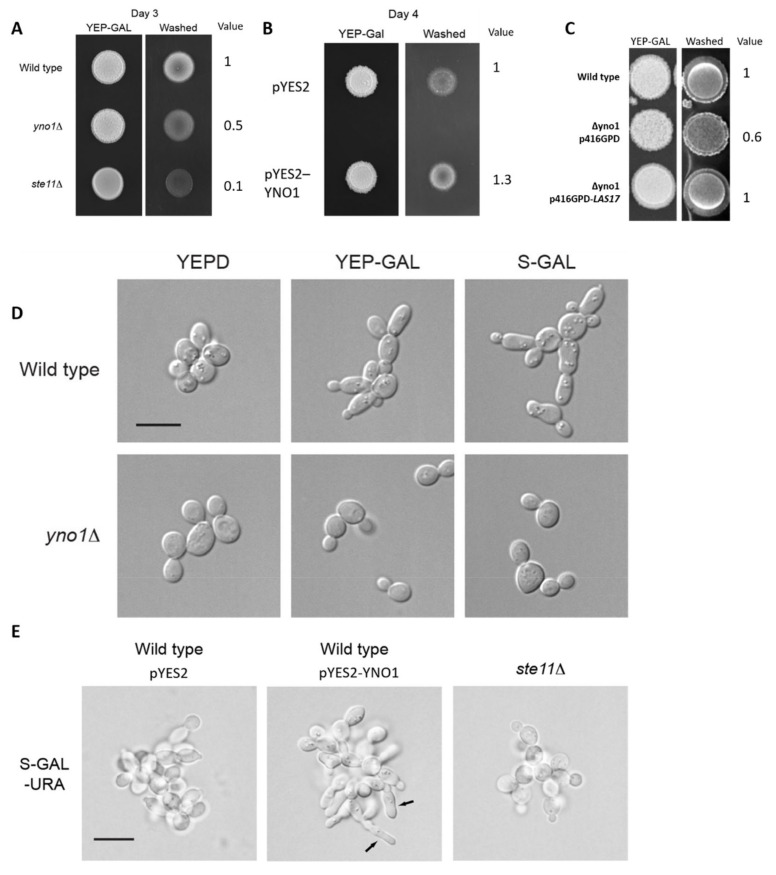
Evaluating the role of Yno1p in regulating filamentous growth. (**A**) Wild type cells (PC538), the ∆*yno1* mutant (PC7010), and the *∆ste11* mutant (PC3861) in the ∑1278b strain background were spotted onto YEP-GAL media for 3 days at 30 °C. The plate was photographed, washed in a stream of water, and photographed again. (**B**) Wild type Σ1278b cells (PC538) harboring pYES2 or pYES2-*YNO1* plasmids were grown on SC-Ura to maintain selection for the plasmids and spotted onto YEP-GAL media and incubated for 4 days at 30 °C. The plate was photographed, washed in a stream of water and photographed again. (**C**) Wild type cells and the ∆*yno1* mutant either harboring p416GPD or p416GPD-*LAS17* plasmids after selection on SC-Ura were spotted onto YEP-GAL media incubated for 4 days at 30 °C. The plate wash assays (PWA) in (**A**–**C**) was repeated in multiple biological replicates and invasive growth was quantified using ImageJ. In (**A**), wild type invasion was set to a value of 1.0, which varied by ± 0.05 across trials. The invasive growth of the ∆*yno1* mutant was 0.55 ± 0.11, and ∆*ste11* was 0.1 ± 0.09 (N = 3, *p* < 0.03). In (**B**), wild type invasion was set to a value of 1.0, which varied by ± 0.12 across trials. The invasion of pYES-YNO1 was 1.42 ± 0.14 (N = 3, *p* < 0.01). In (**C**), the intensity of the spots after the PWA are indistinguishable between the wild type p416GPD and ∆*yno1* p416GPD-*LAS17* cells and are reduced to 0.8 +/– 0.1 in the ∆*yno1 p416GPD* mutant (N = 4; *p* = 0. *p* = 0.05). (**D**) Wild type cells (PC538) and the ∆*yno1* mutant (PC7010) were grown to mid-log phase in YEPD, YEP-GAL, or S-GAL media and examined by differential interference contrast (DIC) microscopy at 100X magnification. Bar, 10 microns. (**E**) Analysis of cells overexpressing *YNO1* by the single cell invasive growth assay (Cullen and Sprague, 2000). Wild type cells (PC538) cells harboring pYES2 or pYES2-*YNO1* were grown to saturation in liquid culture and spread onto SC-Ura medium. A spot of galactose (10 microliters of 20% galactose) was spotted onto the plate, which was incubated for 16 h at 30 °C. Cells were examined by DIC microscopy on plates at 100X. Bar, 10 microns. Arrows mark several examples of hyper-polarized cells.

Filamentous growth is accompanied by an elongated morphology that results from a delay in the G1 and G2-phases of the cell cycle, a change in cell polarity, and enhanced cell–cell adhesion mediated by the adhesion/flocculin Flo11p. Collectively, these and other changes promote increased invasion of cells into substrates [27,74]. Due to the fact that filamentous growth is strictly dependent on Flo11p [77], and endogenous *FLO11* expression is low in BY4741 [78], the filamentous strain background (Σ1278b) was chosen for further experiments [46]. The *YNO1* gene was disrupted in an otherwise wild type filamentous strain by homologous recombination of a PCR-amplified *KlURA3* cassette containing primer sequences to direct integration at the *YNO1* locus. As a first test, the plate-washing assay [79] was performed to test the role of Yno1p in filamentous growth. Yeast cells can penetrate semi-solid agar media and are resistant to removal from agar plates by rinsing with water, by an assay called the plate-washing assay [22]. By this test, the ∆*yno1* strain was defective for invasive growth (Figure 7A).

The invasive growth defect of the ∆*yno1* strain was less severe than a strain lacking a functional MAP kinase cascade (∆*ste11* strain). The effects on cell morphology were also examined. Galactose-induced filamentous growth resulted in the formation of elongated cells in the wild type strain but not the ∆*yno1* mutant (Figure 7D). Cells overexpressing *YNO1* were also examined. Wild type cells of the Σ1278b background were transformed with a control plasmid, pYES2 or a plasmid that overexpresses *YNO1,* pYES2-YNO1 (for details see [5]). Compared to the strain harboring the empty vector (pYES2), the *YNO1* overexpression construct (pYES2-*YNO1*) improved invasive growth (Figure 7B). The single cell invasive growth assay, which provides information about budding pattern and cell elongation [49], was also performed. Under conditions that induce filamentous growth (10 µL of 20% galactose plated on a SC-Ura medium), cells overexpressing *YNO1* showed hyper-polarized cell morphologies (Figure 7E, arrows).

Based on the phenotypes on wiskostatin discussed above, we wanted to test if Yno1p might impact actin polymerization during filamentous growth and whether hydrogen peroxide plays some role. The *LAS17* ORF was cloned into the vector p416GPD, which presumably results in high levels of the N-WASp homologue. This construct was introduced into wild type cells and the ∆*yno1* mutant in the ∑1278b strain background. Here, Las17p overexpression restored invasive growth of the ∆*yno1* mutant (Figure 7C).

**Figure 8:**
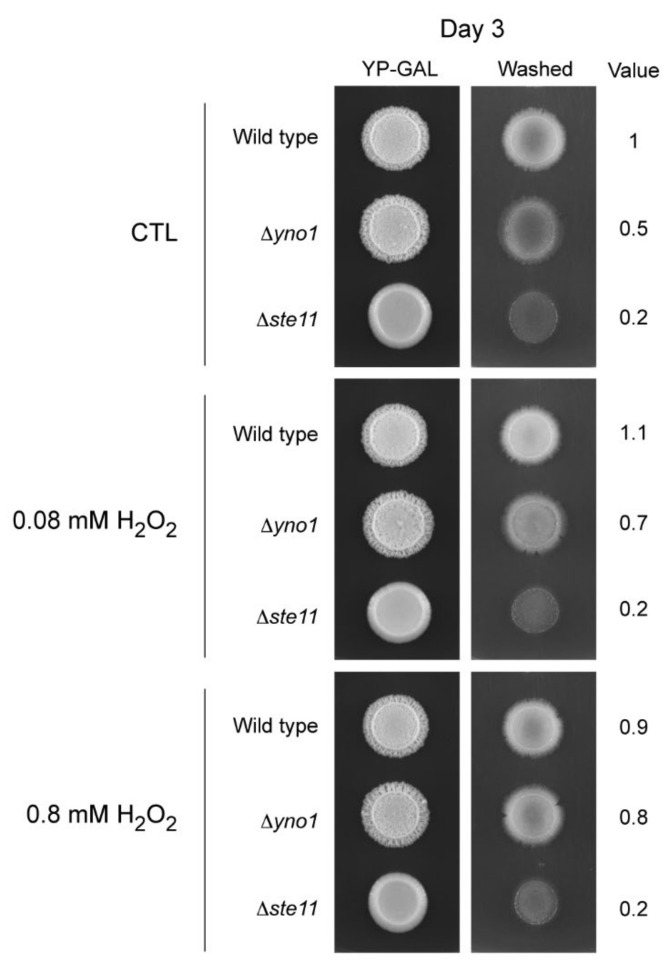
The effect of hydrogen peroxide on invasive growth of the ∆*yno1* mutant alongside control strains. Wild type cells (PC538), the ∆*yno1* mutant (PC7010) and the *∆ste11* mutant (PC3861) in the ∑1278b strain background were spotted onto YEP-GAL media containing the indicated concentrations of H_2_O_2_ for 3 days at 30 °C. Plates were photographed, washed, and photographed again. Invasive growth was quantified by ImageJ analysis.

Previously, we have shown that the ∆*yno1* mutant has less stable F-actin cables. Addition of hydrogen peroxide promotes actin nucleation and can compensate for the loss of Yno1p [5]. We found that low doses of hydrogen peroxide (0.08 mM H_2_O_2_) improved the invasive growth of the ∆*yno1* strain (Figure 8). This result supports the idea that reduced superoxide/hydrogen peroxide underlies the invasive growth defect of the ∆*yno1* mutant. Higher doses of hydrogen peroxide (0.8 mM H_2_O_2_) further improved invasive growth. In a ∆*ste11* mutant, addition of hydrogen peroxide had no effect. Therefore, Yno1p-dependent control of ROS is a component of the filamentous growth response in yeast.

### 3.5. Yno1p, Actin Polymerization and Filamentous Growth

**Figure 9:**
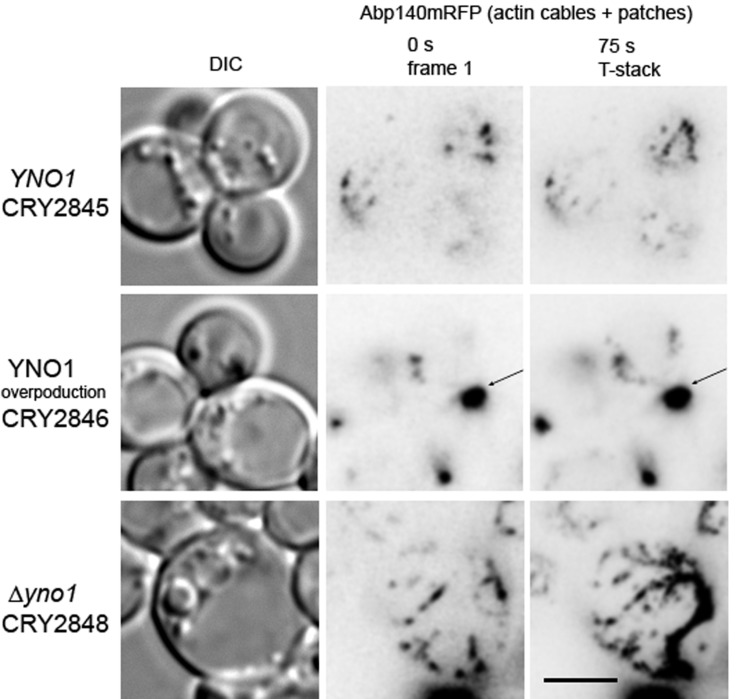
The effect of Yno1p on F-actin distribution at the cell cortex. F-actin structures were visualized by expression from an Abp140-mRFP fusion protein that is integrated into the chromosome. The strains CRY 2845 (MATa SY3089 ste4 FUS1-lacZ FUS1-HIS3 ura3-52; pYES ABP140-RFP-T rho0), CRY 2846 (MATa SY3089 ste4 FUS1-lacZ FUS1-HIS3 ura3-52; pYES YNO1 ABP140-RFP-T rho0), and CRY 2848 (MATa SY3089 ste4 FUS1-lacZ FUS1-HIS3 ura3-52; yno1::KIURA3 ABP140-RFP-T rho-) were grown in synthetic SC-Ura medium and analyzed after addition of 3% galactose for 7 h. Dynamics of F-actin structures was expressed as a T-stack (superposition of 15 images of the cortical F-actin taken in 5 sec intervals). For each strain at least 3 biological and more than 100 cells were inspected. In the strain CRY2845 small actin patches were observed, whereas in the strain CRY2848 there was a prevalence for cortical actin cables. In strain CRY2846, static F-actin bodies of large size are frequently formed (arrow). Bar, 5 μm.

We also tested the effect of Yno1p on the F-actin system during filamentous growth using a collection of rho0 Σ1278b strains expressing Abp140mRFP from chromosomal sites. To obtain exponentially growing cells for investigation, we grew the strains in glucose-containing synthetic media (SC-Ura) to which 20% galactose was added (up to 3%). Galactose induced not only the invasive growth in wild type rho0 strains, but also led to an overproduction of Yno1p in a particular strain harboring an extra copy of YNO1 on the vector pYES2. Since the F-actin system is highly dynamic, we analyzed the rearrangement of Abp140mRFP in corresponding strains at a single cortical layer in time (Figure 9).

We found that wild type cells display a dynamic system of actin patches and filaments. Similar to WT cells, the ∆*yno1* cells did not display a loss of cortical actin cables. These cells were usually enlarged, and their enlargement was associated with an increased number of cortical actin cables, and their increased dynamics. The enlarged and round morphology of the ∆*yno1* mutant might account for its invasive growth defect. In contrast, the overproduction of Yno1 in this genetic background cells led to a subsequent accumulation of F-actin packages moving along the cortical actin filaments (see Supplementary Movies). Our experiments suggest that the localized activity of Yno1p is required for stabilization of cortical bulk of actin, which would be expected to promote the increase in cell polarization that occurs during filamentous growth.

## 4. Discussion

A longstanding and popular paradigm has been that ROS are detrimental for the cell. In 1956, Denham Harman proposed his famous “Free Radical of Aging Theory” [80]. In this pioneering publication, it was proposed that irradiation of cells resulted in the emergence of ROS thus leading to cellular damage, DNA mutations, cancer, and finally aging. By the turn of the century, this theory was challenged by several key discoveries. Mitohormesis describes a phenomenon in which impaired glycolysis leads to a stimulation of mitochondrial respiration attributed with an increased ROS production. Surprisingly, this surplus of ROS resulted in the increase of lifespan in the model worm *C. elegans* [81]. The “aging-ROS” contradiction is the focus of a well-written review [82]. It is now becoming increasingly clear that ROS, although detrimental in some settings, can also act as important signaling molecules.

In fungi, both ROS and NADPH oxidases fulfill important signaling functions that mainly contribute to cellular differentiation. ROS generation is connected to filamentous growth by several different mechanisms [83,84,85], although the mechanisms by which ROS generation is regulated remains incompletely explored. In filamentous fungi, such as *Aspergillus nidulans*, *Podospora anserina*, and *Neurospora crassa*, NADPH oxidases and, thus, ROS are involved in the development of fruiting bodies (cleistothecia) [86,87,88,89]. In the grass endosymbiont *Epichloe festucae*, NADPH oxidases are needed for asexual spore formation and polarized growth including the formation of hyphae [90]. The NADPH oxidase NoxA functions in a complex with the NOX regulator NoxR. NoxR fulfills the function of a scaffold that establishes contact to BemA and Cdc24 (a GEF for Cdc42) that are essential for polarized growth [90].

These observations are consistent with our findings in *S. cerevisiae*. It was previously demonstrated that the yeast NADPH oxidase Yno1p is involved in both the stabilization of Yck1/2p and localization of Ras2p [8,15]. In some settings, Ras2p has been shown to regulate the small (monomeric) Rho-like GTPase Cdc42, which is a “master regulator” of polarized growth [31]. Deduced from several synthetic lethality screens and loss-of-function studies a genetic interaction between Bem2p/Cdc42p and Yck1/2p can also be speculated [91,92,93]. In fact, we observed a genetic interaction between Bem2p (one of the GAPs for Cdc42p [94]) and Yno1p. The *∆bem2* *∆yno1* double mutant was hypersensitive to wiskostatin, an F-actin inhibitor. In general, most phenotypes of Yno1p are deeply interwoven with the nucleation of actin. Cdc42p mediates actin polymerization via a complex interplay between the Las17p/Vrp1p dimer, and the Arp2/3 complex [95].

Deletion of *YNO1* increased the sensitivity to wiskostatin in a *∆vrp1* or *∆las17* background. This result indicates that Yno1p activity and Las17p/Vrp1 probably act in parallel pathways during regulation of the actin cytoskeleton. However, we could also observe that a double deletion of *LAS17* and *VRP1* is synthetic lethal.

During osmotic stress, the actin cytoskeleton is also reorganized [40]. We show that addition of 1 M NaCl induced the transcription of the *YNO1* gene, which was dependent on the transcription factor Msn2p. Vacuolar fragmentation, which occurs in response to osmotic shock and is dependent on actin polymerization [42,96], was more severe and lasted for a longer time in the *∆yno1* mutant. During osmotic stress, Yno1p interacts with Sod1p to produce hydrogen peroxide. A *∆yno1* *∆sod1* double mutant shows an increased wiskostatin sensitivity compared to the respective single gene deletions. Recently, it was demonstrated that vacuoles in a *∆sod1* background show elevated vacuolar fragmentation [97]. Experiments in the plant *Arabidopsis thaliana* demonstrate that the importance of ROS as second messengers in response to hyperosmotic shock are evolutionary conserved [98]. Our data also indicate that Yno1p via the activation of Cdc42p regulates its own transcription, actin polymerization and osmotic stress response in parallel. Such a feed forward situation leads to an increase in Yno1p activity and the resulting burst of ROS would lead to cellular damage. Such a system could work well if there is also a mechanism of deactivation after the adaptation phase. This particular positive feedback loop is eventually stopped by the ERAD as previously demonstrated for Yno1p [15]. Eventually also catalases are involved in the removal of a H_2_O_2_ excess and thus “over signaling”.

**Figure 10:**
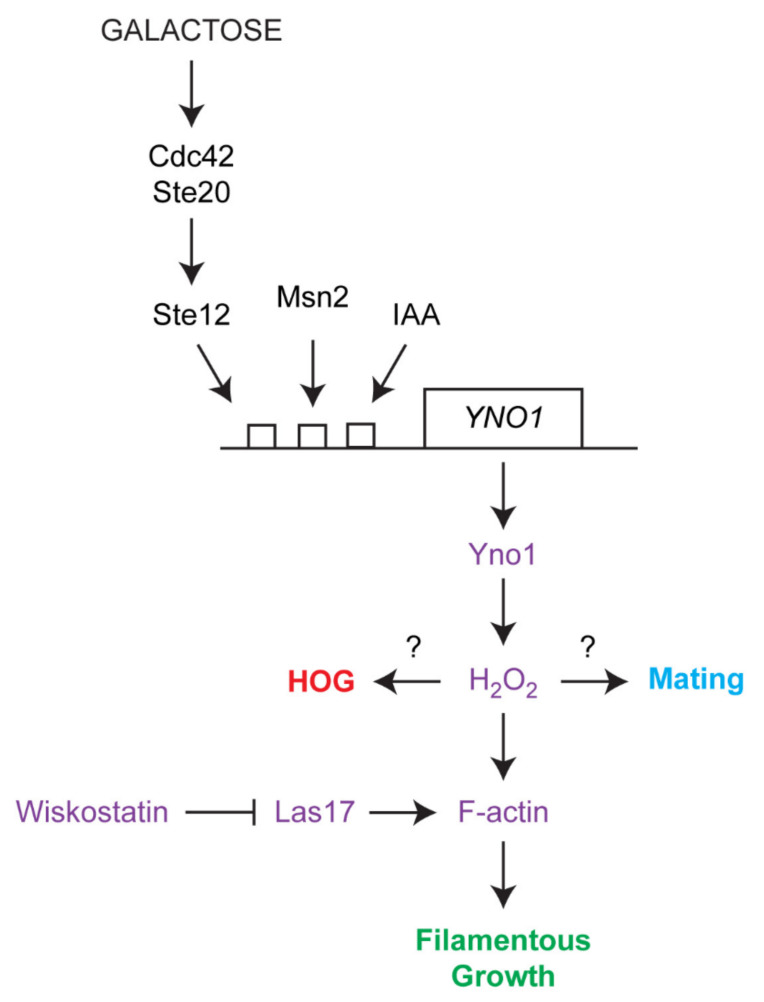
Model for the action Yno1p during filamentous growth. Galactose induces filamentous growth. The transcription factors Ste12p and Msn2p are activated via Cdc42p/Ste20p and promotes the expression of the Yno1p gene. IAA treatment induces filamentous growth and *YNO1* expression independent of Ste12p. Yno1p in an interplay with Sod1p produces hydrogen peroxide that is involved in pheromone response (blue letters), osmotic stress response (red letters) and filamentous growth (green letters). An additional target of Yno1p-derived ROS is the actin assembly factor Las17p that promotes the nucleation of F-actin (purple letters). Our data indicate that stabilized F-actin may impact filamentous/invasive growth.

The actin cytoskeleton is also reorganized during morphogenetic responses that are controlled by signal transduction pathways. One example is filamentous/invasive growth, which is controlled by many pathways, including a Cdc42p-dependent MAPK pathway, and which involves reorganization of the actin cytoskeleton (Figure 10) [20]. Fusel alcohols and non-preferred carbon sources that induce invasive growth [27] were also found to induce expression of *YNO1*. Interestingly, deletion of one of the transcription factors that regulates the fMAPK pathway, *STE12*, reduced the transcriptional induction of *YNO1* to galactose, but not to IAA (Figure 10). Therefore, the expression of *YNO1* might be induced by multiple triggers of filamentous/invasive growth.

Yno1p was required for invasive growth, which might result from hydrogen peroxide production, which leads to altered actin filament assembly. Addition of H_2_O_2_ improved invasive growth in cells lacking Yno1p. Moreover, overexpression of Las17p complemented the invasive growth defect seen in cells lacking Yno1p (Figure 10). Time-lapse microscopy of Abp140mRFP-labeled cells revealed that overexpression of Yno1p induced formation of F-actin bodies in a similar way as described previously by increased concentration of added H_2_O_2_ [99]. These stabile actin aggregates resemble oxidized actin bodies, which may contain various actin-binding proteins. The question remains how the formation of these F-actin structures is related to invasive growth of this particular strain. A role for NADPH oxidases in the regulation of filamentous growth may be conserved among fungi. The NADPH oxidase FRE8 *in C. albicans* leads to a burst of ROS that is a prerequisite for morphological changes during invasive growth and pathogenicity [6]. Although the closest homologue to Fre8p *in C. albicans* is not Yno1p but Fre3p in *S. cerevisiae*, an overexpression of Fre3p in *S. cerevisiae* did not cause superoxide production, at least under the conditions tested [5]. These findings show that exploring this family of proteins will have value in understanding the role of ROS in fungal dimorphism.

Actin reorganization also occurs during mating projection formation, which is also controlled by a related Cdc42p-dependent MAP kinase pathway. Treatment of Mata cells with alpha factor at levels above physiological concentrations induce apoptotic cell death attributed with a burst of ROS [34]. This could be a trigger to remove cells after unsuccessful mating from the population [100]. In the present work, we show that the ROS is derived from the yeast NADPH oxidase. In the control strain, a~3-fold increase in superoxide levels was detected, whereas in a ∆*yno1* strain no such increase was observed. It is assumed that low levels of ROS promote cell survival, whereas high doses of ROS initiate apoptosis [34,101]. Previously, we showed that overexpression of Yno1p resulted in a~9-fold increase in cellular superoxide levels. These high ROS levels correlate with an increased apoptosis and necrosis. Deletion of the yeast metacaspase Yca1p that is an essential component of the yeast apoptotic program diminished the increased cell death that was induced by *YNO1* overexpression. This result demonstrates that ROS *per se* are not detrimental for the cells, but rather fulfill signaling functions depending on location and concentration of ROS. Although the increase of ROS in the ∆*yno1* background after addition of alpha factor was not observed, the incidence of apoptosis was still high. In the control strain as well as ∆*yno1* strain an unphysiological surplus of alpha factor reduced cell survival to less than 50%. This result indicates that ROS originating from the yeast NADPH oxidase Yno1p fulfills signaling functions independent on the induction of apoptosis and polarized growth/shmooing.

## 5. Conclusions

Branched actin filaments form at sites of polarized growth and their nucleation is dependent on Las17p/Vrp1p as well as the Arp2/3 complex. In yeast cells, the morphological manifestations of this branched F-actins are so called “actin patches” that have to be clearly distinguished from actin cables. In growing cells, these actin patches mark the site of bud emergence and appear afterwards in the daughter cell [102]. Under specific conditions, actin patches also accumulate at the shmoo tip of mating cells and at the distal tip of pseudohyphal cells and are required for the morphological changes of these cells [20,33]. By comparison, during osmotic stress actin cables are lost and patches redistribute from the daughter to mother cells [103]. A correct localization of these patches at sites of polar growth and in the cortical regions of the cell is mediated by the plasma membrane bound G-protein Cdc42p by a recruitment of Las17p/Vrp1 and Arp2/3 [104]. In the current work, we show that a yeast NADPH oxidase (Yno1p) is involved in this process. Yno1p produces superoxide that is instantly dismutated into hydrogen peroxide by the superoxide dismutase Sod1p [8]. This ROS acts as a signaling molecule and seems to modulate actin functions through Las17p as shown by screens based on the drug wiskostatin, a chemical inhibitor of F-actin polymerization. Accordingly, we could demonstrate that Yno1p overexpression leads to larger large actin patches, whereas in the corresponding deletion background the prevalence for actin cables during filamentous growth is increased. Besides its role in actin nucleation, Cdc42p is a central regulator located upstream of several MAPK pathways that modulate the response during filamentous growth, osmotic stress and mating (Figure 1). In a ∆*yno1* deletion strain, reduced ROS levels occur, which presumably underlie the defects in filamentous growth and recovery of vacuolar morphology upon a hyperosmotic stress. The complexity of this regulatory network is further evident by the fact that transcription factors that are controlled by these MAPK pathways stimulate the expression of the *YNO1* gene. Further studies in the emerging field of ROS signaling in *Saccharomyces cerevisiae* may continue to provide information about this type of signaling response.

## Data Availability

Data is contained within the article or supplementary material.

## Supplementary Materials

The following are available online at https://www.mdpi.com/2076-3921/10/2/322/s1, Figure S1: GFP-based *YNO1*-reporter; Supplementary Material (Strains (including construction of the GFP-based *YNO1*-GFP reporter); media composition; and primer sequences); Supplementary movies (CRY2845; CRY2846; CRY2848).

## Abbreviations

ER: endoplasmic reticulum; HOG: hyperosmolarity glycerol response; IAA: isoamyl alcohol; PAK: serine/threonine p21 activated kinase; MAP: mitogen-activated protein; MAPK: mitogen-activated protein kinase; MAPKK: mitogen-activated protein kinase kinase; MAPKKK: mitogen-activated protein kinase kinase kinase; ROS: reactive oxygen species; DIC: Differential interference contrast; NADPH: Nicotinamide adenine dinucleotide; DHE, dihydroethidium; YEPD: yeast extract peptone dextrose; SDS: sodium-dodecyl-sulfate (SDS); PAGE: polyacrylamide gel electrophoresis: PWA: Plate wash assays.
